# Rubber Leaf Disease Recognition Based on Improved Deep Convolutional Neural Networks With a Cross-Scale Attention Mechanism

**DOI:** 10.3389/fpls.2022.829479

**Published:** 2022-02-28

**Authors:** Tiwei Zeng, Chengming Li, Bin Zhang, Rongrong Wang, Wei Fu, Juan Wang, Xirui Zhang

**Affiliations:** ^1^School of Information and Communication Engineering, Hainan University, Haikou, China; ^2^Mechanical and Electrical Engineering College, Hainan University, Haikou, China

**Keywords:** rubber leaf disease recognition, lightweight neural network, attention mechanisms, GMA block, GMA-Net

## Abstract

Natural rubber is an essential raw material for industrial products and plays an important role in social development. A variety of diseases can affect the growth of rubber trees, reducing the production and quality of natural rubber. Therefore, it is of great significance to automatically identify rubber leaf disease. However, in practice, different diseases have complex morphological characteristics of spots and symptoms at different stages and scales, and there are subtle interclass differences and large intraclass variation between the symptoms of diseases. To tackle these challenges, a group multi-scale attention network (GMA-Net) was proposed for rubber leaf disease image recognition. The key idea of our method is to develop a group multi-scale dilated convolution (GMDC) module for multi-scale feature extraction as well as a cross-scale attention feature fusion (CAFF) module for multi-scale attention feature fusion. Specifically, the model uses a group convolution structure to reduce model parameters and provide multiple branches and then embeds multiple dilated convolutions to improve the model’s adaptability to the scale variability of disease spots. Furthermore, the CAFF module is further designed to drive the network to learn the attentional features of multi-scale diseases and strengthen the disease features fusion at different scales. In this article, a dataset of rubber leaf diseases was constructed, including 2,788 images of four rubber leaf diseases and healthy leaves. Experimental results show that the accuracy of the model is 98.06%, which was better than other state-of-the-art approaches. Moreover, the model parameters of GMA-Net are only 0.65 M, and the model size is only 5.62 MB. Compared with MobileNetV1, V2, and ShuffleNetV1, V2 lightweight models, the model parameters and size are reduced by more than half, but the recognition accuracy is also improved by 3.86–6.1%. In addition, to verify the robustness of this model, we have also verified it on the PlantVillage public dataset. The experimental results show that the recognition accuracy of our proposed model is 99.43% on the PlantVillage dataset, which is also better than other state-of-the-art approaches. The effectiveness of the proposed method is verified, and it can be used for plant disease recognition.

## Introduction

The rubber tree is one of the most important economic crops in the tropics, and the planting area of rubber trees in China is more than 1.16 million hectares, more than half of which are planted in Hainan Province (Ali et al., 2020; Li and Zhang, 2020). The milky latex extracted from the tree is the primary source of natural rubber, which is an essential raw material for industrial products. However, rubber leaf diseases cause annual losses of approximately 25% of the total yield of natural rubber and cause significant economic losses. A variety of diseases can affect the growth of rubber trees, reducing the production of natural rubber and seriously hindering the development of the natural rubber industry. Hence, the identification and diagnosis of rubber leaf diseases (e.g., powdery mildew disease, rubber tree anthracnose, periconla leaf spot disease, and Abnormal Leaf Fall Disease) are of great significance for increasing the yield of natural rubber and have received extensive attention from rubber planting workers and experts on disease and pest control. Unfortunately, manual identification and diagnosis are time-consuming and laborious in practice, and the recognition accuracy does not satisfy the requirement.

To solve the problems caused by the manual diagnosis, researchers have proposed some machine learning-based methods for plant disease recognition (Sladojevic et al., 2016; Hu et al., 2018). The plant disease recognition method based on traditional machine learning is mainly through the manual design of classification features, such as color features (Semary et al., 2015), shape features (Parikh et al., 2016), texture features (Mokhtar et al., 2016), or the fusion of two or more manual features (Shin et al., 2020). However, the manual features in these approaches are selected based on human experience, which limits the generalizability of the models.

Recently, deep convolutional neural networks (DCNNs) have been widely applied in image and video classification tasks (Ren et al., 2020). Compared with traditional machine vision algorithms, DCNN can complete feature extraction and classification tasks through the self-learning ability of the network without manual design features (Liu et al., 2017). Anagnostis et al. (2020) offered a Walnut disease classification system using CNN with an accuracy range from 92.4 to 98.7%. Zhu et al. (2019) investigated a two-way attention model for plant recognition and validated the method in four challenging datasets, and the recognition accuracy reaches 99.8, 99.9, 97.2, and 79.5%, respectively. Anwar and Anwar (2020) used DenseNet networks without transfer learning methods to identify four different citrus diseases, and experimental results show that the model can accurately treat citrus diseases, with an accuracy of 92% on the given test dataset. Suh et al. (2018) proposed a transfer learning classifier based on the VGG-19 CNN architecture for the classification of sugar beet and volunteer potato and reported a maximum of 98.7% accuracy for the classification. Maeda-Gutiérrez et al. (2020) classified nine different types of tomato diseases and a healthy class using AlexNet, GoogleNet, InceptionV3, and ResNet18, and the highest recognition rate reached 99.12%. According to these studies, DCNN has higher predictive value and reliability than well-trained humans.

To run the DCNN model on mobile and embedded devices, some scholars have also proposed lightweight networks, which have the advantages of fewer parameters and smaller model size, such as MobileNetV1 (Howard et al., 2017), MobileNetV2 (Sandler et al., 2018), ShuffleNetV1 (Zhang et al., 2018), and ShuffleNetV2 (Ma et al., 2018). Liu et al. (2020) proposed a robust CNN architecture for the classification of six different types of grape leaf disease. This method uses depth-separable convolution instead of standard convolutional layers to reduce model parameters, and the recognition accuracy reached 97.22%. Rahman et al. (2020) proposed a two-stage small CNN architecture named SimpleNet for rice diseases and pest identification with an accuracy of 93.3%. This method is fine-tuned based on VGG16 and InceptionV3 structure to reduce model parameters. The parameters of this network model are less than those of classical CNN models. Tang et al. (2020) identified grape disease image based on improving the ShuffleNet architecture, with an accuracy of 99.14%, similar to the existing CNN models, but the computational complexity is slightly lower. These studies have shown good results, but different diseases have complex morphological characteristics of disease spots at different stages and scales, and the same scale often has similar characteristics, which makes image disease recognition difficult. Therefore, how to fully extract the key information of the local area is the key to improve the performance of disease image recognition. To address these issues, many researchers have focused on attentional features of mechanism-based methods. Li et al. (2020) used the GoogleNet model and embedded SENet attention mechanism to enhance information expression of Solanaceae diseases, with an accuracy rate of 95.09%, and the model size is 14.68 MB, which can be applied to the mobile terminal to identify Solanaceae disease. Mi et al. (2020) proposed a novel deep learning network, namely, C-DenseNet, which embeds convolutional block attention module (CBAM) in the densely connected convolutional network with an accuracy rate of 97.99%. Wang et al. (2021) proposed a novel lightweight model (ECA-SNet) based on Shufflenet-V2 as the backbone network and introduced an effective channel attention strategy to enhance the model’s ability to extract fine-grained lesion features with an accuracy rate of 98.86%. Chen et al. (2021) chose the MobileNet-V2 as the backbone network and added the attention mechanism to learn the importance of interchannel relationships and spatial points for input features, and the average accuracy reaches 98.48% for identifying rice plant diseases. In addition, to further improve the performance of feature extraction, some work improves the representation of feature information by integrating multiple-scale features (Liu et al., 2018; Zhang et al., 2020; Pan et al., 2021). Shen et al. (2021) proposed a feature fusion module named adaptive pyramid convolution, which aggregates the features of different depths and scales to suppress the messy information in the background and enhance the feature representation capability of local regions. Sagar (2021) proposed to enhance the dependence between local features and global features by extracting spatial and channel attention features in parallel. Although these methods achieve good results, they can easily increase computational complexity.

Inspired by the above research, we proposed a deep neural network model, namely, group multi-scale attention network (GMA-Net). The main innovations and contributions are summarized as follows:

(1) A rubber leaf disease dataset is established, and the image data augmentation scheme is used to synthesize new images to diversify the image dataset and enhance the anti-interference ability under complex conditions.
(2) The model uses group convolution structure to reduce model parameters and provide multiple branches for multi-scale feature extraction, then embeds dilated convolution to improve the model’s adaptability to the scale variability of disease spots, and adds a cross-scale attention feature fusion (CAFF) module to suppress complex background information to strengthen the disease features fusion at different scales.

The rest of this article is organized as follows. The “Materials and methods” section presents the dataset and methods adopted in this study. The “Experimental results and analysis” section presents the experiments for evaluating the performance of the model and analyzes the results of the experiments. Finally, the “Conclusion and future work” section summarizes the main conclusions and future avenues.

## Materials and Methods

### Dataset Preparation

#### Data Acquisition

The spread of rubber leaf disease is closely related to season, temperature, light, and other factors. For example, powdery mildew disease mainly occurs in spring, and it is more likely to breed disease after rainy days. The rubber leaf disease dataset is created, which included 2,788 rubber leaf samples collected from the rubber tree cultivation farm of Rubber Research Institute, Chinese Academy of Tropical Agricultural Sciences in Danzhou City, Hainan Province, in April 15–20, 2021, and May 13–16, 2021. The types of rubber leaf diseases of these samples were known in advance and labeled according to the domain experts’ knowledge. The classification and labeling of different rubber leaf diseases only consider different external visual symptoms, and then image data were captured in the laboratory. Red, green, and blue (RGB) leaf images were taken with the default parameters of the NIKON D90 camera (with a lens Tamron AF 18–200 mm f/3.5–6.3) and iPhone 11 mobile phone. A total of 5 types of image samples of rubber leaves were collected, including four kinds of diseases (i.e., powdery mildew disease, rubber tree anthracnose, periconla leaf spot disease, and abnormal leaf fall disease) and healthy leaves.

**FIGURE 1:**
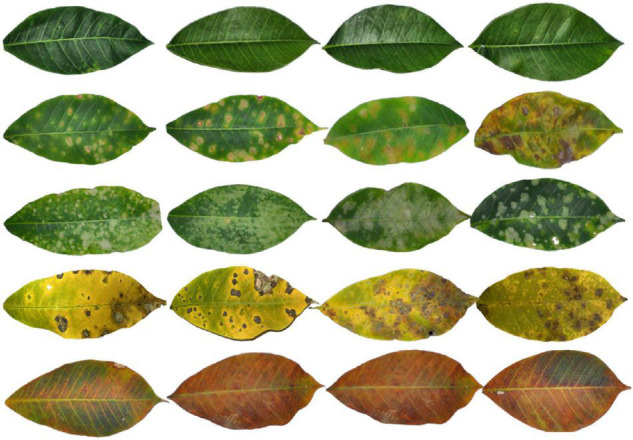
Sample images of our constructed rubber dataset, from top to bottom, are healthy leaves, powdery mildew disease, rubber tree anthracnose, periconla leaf spot disease, and abnormal leaf fall disease.

Examples of typical symptoms of these rubber leaf diseases are given in Figure 1. Healthy rubber leaves appear green, the surface is smooth without disease spots, and the veins are visible. Powdery mildew disease is considered one of the major diseases that threaten the stability of natural rubber production. It spreads rapidly because the pustules can be dispersed for miles on air currents. The lesions initially appear as small, radiating silver-white spots of cobweb-like hyphae scattered on the surface or back of the leaf and then develop to the entire leaf. As the lesion matures, the powdery mildew spots turn into white ringworm-like spots, the surface of the leaves becomes dried and yellow, and finally falls off. The powdery mildew disease can cause high yield losses when severe epidemics occur. Rubber tree anthracnose can appear on stalks, leaves, petioles, tender shoots, or fruits of the rubber tree. The symptoms of this disease begin at the tip and edge of the leaf and can be observed on the leaf as yellow or brown water-stained spots, while as the lesion matures, it becomes irregular, narrow, and gray-white. Periconla leaf spot disease appears as small, dark brown spots scattered on the leaf surface, the tissues at the center of the lesions later decay and become gray to white with black rings at the margin, and the lesions are oval to circular spots, with 0.2–4 cm in diameter. For abnormal leaf fall disease, the small dark brown water-stained spots on the leaf blade may have light brown halos; as the lesions mature, they expand to circular or nearly circular lesions with a diameter of 1–3 mm and turn dark brown near the stalk of the leaf when some of the lesions appeared perforated.

#### Data Augmentation

Image preprocessing was carried out on the RGB raw images before image data augmentation, including image scaling, image clipping, and image background removal. Then, the dimensions of the sample images were uniformly resized to 224 × 224 pixels as input to image analysis to reduce the computational cost and improve the image processing efficiency. Our constructed dataset contains 885 images of powdery mildew disease, 829 images of rubber tree anthracnose, 335 images of periconla leaf spot disease, 521 images of abnormal leaf fall disease, and 218 images of healthy leaves. By analyzing the distribution of the number of samples in each category, the dataset we construct is unbalanced. Therefore, the image data enhancement scheme is used to synthesize new images to diversify the image dataset, suppress the impact of unbalanced data, and enhance the anti-interference ability under complex conditions. In this article, based on the Keras’ framework, the batch size is set to 32, and brightness adjustment, rotation, scaling, horizontal flip, vertical flip, and other methods are selected to synthesize new images to diversify the image dataset. The specific image augmentation operation is shown in Table 1. It should be noted that the data enhancement method adopted in this article will not reduce the size of the image, nor will it change the image’s overall color. Finally, the enhanced dataset distribution contains 1,982 images of powdery mildew disease, 2,516 images of rubber tree anthracnose, 2,350 images of periconla leaf spot disease, 2,406 images of abnormal leaf fall disease, and 2,396 images of healthy leaves, and the detailed report of the dataset before and after applying the augmentation process is shown in Table 2.

**TABLE 1:** Parameter set for data augmentation.

Technology	Range
Rescale the image	1./255
Rotation_range	40
Width_shift_range	0.2
Height_shift_range	0.2
Fll_mode	“Nearest”
Horizontal_flip	True
Vertical_flip	True
Brightness_range	(0.6, 0.9)
Zoom_range	(0.5,0.9)

**TABLE 2:** Detailed report of the constructed dataset before and after applying the augmentation process.

Disease name	Class	Images (Raw)	Images (Augmentation)
Healthy leaves	0	218	1982
Powdery mildew disease	1	885	2516
Rubber tree anthracnose	2	829	2350
Periconla leaf spot disease	3	335	2406
Abnormal leaf fall disease	4	521	2396
Total number		2788	11650

### Architectures of Group Multi-Scale Attention Network Model

#### Network Architecture

**FIGURE 2:**
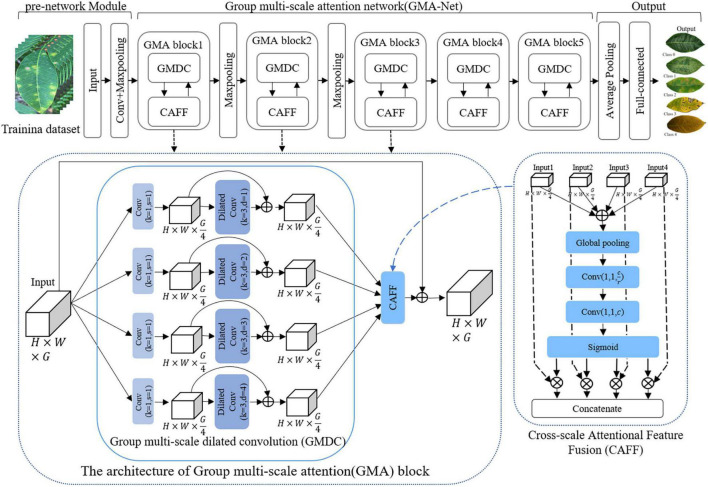
The architecture of the proposed group multi-scale attention network (GMA-Net).

In this article, a GMA-NET model was proposed for rubber leaf disease image recognition. The architecture of the GMA-Net is illustrated in Figure 2. The GMA-Net model includes three parts. The first part is the “pre-network Module” which consists of 3 × 3 convolution layers and max-pooling layers to extract the features of the input image. The second part consists of five cascaded GMA blocks. The GMA block consists of a group multi-scale dilated convolution (GMDC) module and a CAFF module. By utilizing the GMDC module, the network can extract lesion characteristics at different scales and enhance the network’s representation ability. After that, the CAFF module is used to fuse the multi-scale attention feature maps from the output of the GMDC module. The last part is composed of a convolution layer, an average pooling layer, a fully connected layer, and a 5-way Softmax layer. Moreover, the batch normalization layer and ReLu activation function are added after each convolution layer. Overall, the proposed method can effectively extract disease feature representation at different scales and aggregate the cross-scale attention feature, which is conducive to fine-grained disease image classification. We detail the different modules of the network, which are summarized in Table 3.

**TABLE 3:** Detailed architectures of the proposed GMA-Net model in our experiments.

Name	Input	Output	Kernel size	Filter number	Stride
Input	224 × 224 × 3	−	−	−	−
Conv	224 × 224 × 3	112 × 112 × 96	3 × 3	96	2
Map	112 × 112 × 96	56 × 56 × 96	3 × 3	−	2
GMAB 1	56 × 56 × 96	56 × 56 × 64	−	64	−
Map	56 × 56 × 64	28 × 28 × 64	3 × 3	−	2
GMAB 2	28 × 28 × 64	28 × 28 × 128	−	128	−
Map	28 × 28 × 128	14 × 14 × 128	3 × 3	−	2
GMAB 3	14 × 14 × 128	14 × 14 × 192	−	192	−
GMAB 4	14 × 14 × 192	14 × 14 × 208	−	208	−
GMAB 5	14 × 14 × 208	14 × 14 × 256	−	256	−
Avg	14 × 14 × 256	2 × 2 × 256	7 × 7	−	1
Linear	2 × 2 × 256	1 × 1 × 1024	−	−	−
Softmax	1 × 1 × 1024	5	−	−	−

#### Group Multi-Scale Dilated Convolution Module

Different diseases have complex symptoms and morphological characteristics at different stages and scales, and the same scale often has similar characteristics. As shown in Figure 1, the powdery mildew disease has various symptoms, with some appearing scattered cobweb spots and some appearing mass spots. Identifying this disease needs to consider large-scale coarse-grained features (e.g., the size and texture of the lesion). The characterization information of rubber tree anthracnose is similar to periconla leaf spot disease, with relatively yellowish leaves and scattered spots. Small-scale fine-grained features (e.g., color and texture of the lesion) are the key to recognizing these diseases. Therefore, multi-scale information of rubber leaf disease features in the image plays an essential role in accurately identifying the types of rubber leaf disease.

To address these problems, we design a GMDC module, which consists of a group convolution operation and a multi-scale feature extraction operation. Specifically, the purpose of group convolution operation is to reduce parameters and prevent overfitting. The multi-scale feature extraction operation is used to extract multi-scale disease features.

**FIGURE 3:**
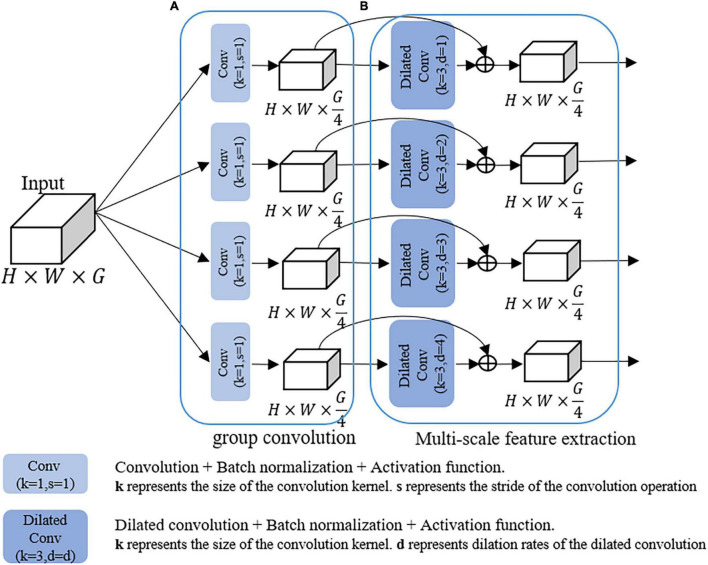
The structure of the GMDC module. **(A)** Multi-branch group convolution. **(B)** Multi-branched dilated convolution with different dilation rates.

As shown in Figure 3, the group convolution structure consists of four parallel 1 × 1 convolutional layers, followed by batch normalization and ReLU activation functions to accelerate network convergence. Multi-scale feature extraction structure extracts multi-scale information through multiple dilated convolutions with different dilation rates, and then skip connections were used to make full use of the relevant information in the feature map. Dilated convolution (Yu and Koltun, 2014) is defined as follows:


(1) (Fl*k)(p)=∑s+lt=pF(s)k(t)

where *F* is a discrete function and *k* is a discrete filter of size (2*r* 1)^2^, ${}_{l}^{*}$ is called a dilated convolution or a *d*-dilated convolution, *k* is a 3 × 3 filter, and the kernel dilation rates are 1–4, respectively.

#### Cross-Scale Attention Feature Fusion Module

**FIGURE 4:**
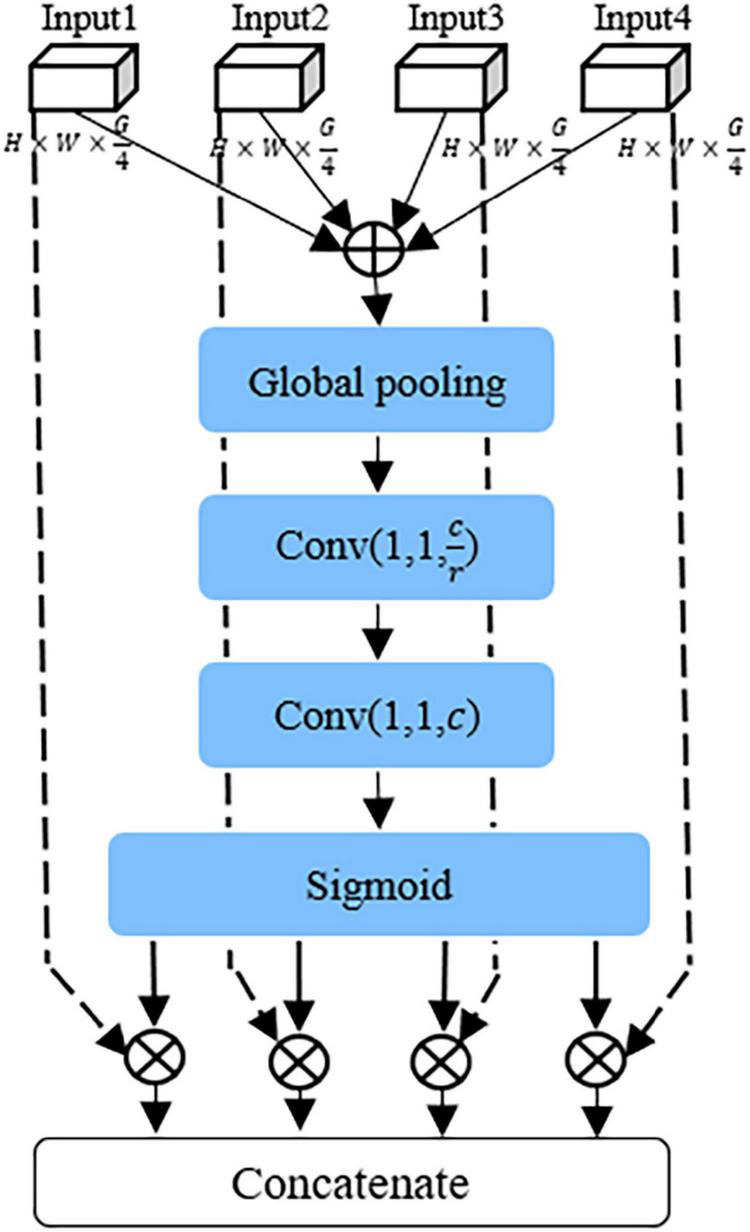
The structure of the cross-scale attention feature fusion module.

Recently, the attention mechanism has been widely used, including image processing (Li et al., 2020; Tang et al., 2020), speech recognition (Xingyan and Dan, 2018), and natural language processing (Bahdanau et al., 2015). The attention mechanism pays attention to the useful information of various channels of the network, inhibits the useless information, which can enhance the representation of disease features, and effectively improves the identification performance of the model. In this study, as shown in Figure 4, a CAFF module was designed to fuse attentional feature maps of different scales.

First, local feature maps of different scales output by GMF module are added point by point to obtain F_*c*_, and then the feature map *F_c_* is compressed into vector Z of 1 × 1 × *C* by the global average pool layer, which can be expressed as follows:


(2) $$F_{c}=Add\left[U_{1}+U_{2}+U_{3}+U_{4}\right]$$

(3) $$Z_{c}=\frac{1}{W\times H}\sum_{i=1}^{W}\sum_{j=1}^{H}U_{c}\left(i,j\right)$$

Then, the global feature *S* is obtained through two fully connected layers, one ReLU activation layer, one batch normalization layer, and one sigmoid layer, respectively. *S* represents the weight coefficient information of different channel features. In this article, 1*1 convolution layer is used instead of fully connected layers to accelerate convergence. The specific formula can be described as:


(4) $$S=\sigma \left(g\left(Z,W\right)\right)=\sigma \left(W_{2}\partial \left(W_{1}Z\right)\right)$$

where σ and ∂ are sigmoid activation function and ReLU activation function, respectively; $W_{1}\in R^{\frac{C}{r}\times C}$ and $W_{2}\in R^{C\times \frac{C}{r}}$ are dimension reduction and restoration parameters, respectively. *r* is the reduction factor, which is set to 16 in this article.

The local feature image output by the GMF module is multiplied point by point with vector *S*, which enhances the feature representation information of diseases at different scales in the input feature map and obtain the local attention information *T*(*x*) representing different scales. *T*(*x*) can be expressed as


(5) $$T\left(U_{i}\right)=Multiply\left(U_{i},S\right)$$

Finally, the local attention information of different scales is connected to generate an effective multi-scale feature descriptor *Y*. *Y* can be expressed as


(6) $$Y=concat\left[T\left(U_{1}\right),T\left(U_{2}\right),T\left(U_{3}\right),T\left(U_{4}\right)\right]$$

The CAFF module can fuse attentional feature maps of different scales to enhance disease information, suppress useless information, and improve model performance.

## Experimental Results and Analysis

### Experimental Configuration and Hyperparameter Setting

Data augmentation and deep learning algorithms are implemented in Keras’ deep learning framework based on CNN using python language. The experimental hardware configurations include an Intel i5-10400F CPU (2.90 GHz), a memory of 16 GB, and an RTX 2060S graphics card.

The enhanced rubber disease dataset and PlantVillage (Hughes and Salathe, 2015) public dataset are divided into three groups, namely, the training set (60%), the validation set (20%), and the test set (20%). Comprehensively considering the performance of hardware devices and training effects, the batch size and the number of iterations for all network models are 16 and 40, respectively, and categorical cross-entropy is used to optimize the model. Stochastic gradient descent (SGD) was adopted for training. The initial learning rate is set to 0.1 for the first epoch, and the learning rate is dynamically adjusted by using the Keras’ ReduceLROnPlatea function. If the accuracy of the validation set does not improve after three iterations, the learning rate will be reduced by half.

### Evaluation Indexes

In this study, precision, recall, F1-score, accuracy, model size, parameters, and floating-point of operations (FLOPs) are selected as evaluation indexes to evaluate the performance of deep learning algorithms comprehensively:


(7) $$Precision=\frac{TP}{TP+FP}$$

(8) $$Recall=\frac{TP}{TP+FN}$$

(9) $$F1sore=\frac{2TP}{2TP+FP+FN}$$

(10) $$Accuracy=\frac{TP+TN}{TN+TP+FP+FN}$$

where TP, TN, FP, and FN are the number of true positive samples, true negative samples, false-positive samples, and false-negative samples, respectively. Precision estimates how many of the predicted positive samples is positive. The recall is the assessment of how many of all positive samples can be correctly predicted as positive. F1-score is the synthesis of precision and recall. Accuracy measures global sample prediction. Model size, parameters, and FLOPs are commonly used to measure model complexity.

### Performance Comparison Between Different Models

**FIGURE 5:**
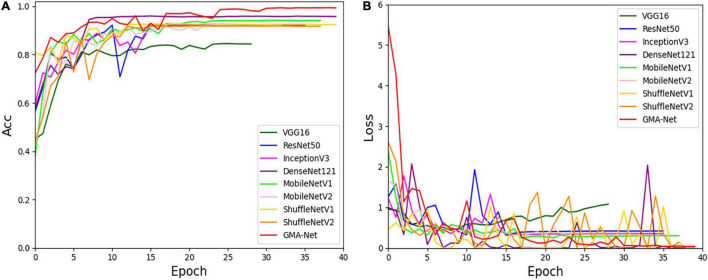
Accuracy curve and loss curve of rubber leaf disease validation set. **(A)** Accuracy curve. **(B)** Loss curve.

To verify the validity of the GMA-Net model, based on our constructed disease dataset, a comparative experiment was carried out with VGG16, ResNet50, GoogleNet, InceptionV3, and DenseNet121 classical CNN models and MobileNetV1, MobileNetV2, and ShuffleNetv2 lightweight models. Moreover, we trained these models according to the training parameters in the “Experimental configuration and hyperparameter setting” section. Figure 5 shows the accuracy curve and loss curve of the above eight networks and GMA-Net on the validation dataset. It can be seen from the accuracy curve and loss curve that GMA-Net has the highest recognition accuracy and quickest convergence rate than other models on the rubber leaf disease dataset, and the model performance is better than the traditional CNN model and lightweight model.

Table 4 compares the nine networks with the precision, recall, F1-score, accuracy, model size, parameters, and FLOP. The GMA-Net model has the best performance, with an accuracy of 98.06%. Model parameters, size, and FLOPs are 0.65, 5.62, and 1.83 M, respectively. The accuracy of VGG16, ResNet50, InceptionV3, and DenseNet121 models is 84.53, 92.61, 92.31, and 96.01%, respectively. Compared with the classical CNN model, the size and FLOPs of our constructed model are ten times smaller, and the accuracy of the proposed GMA-NET is increased by 13.53, 5.45, 5.75, and 2.05%, respectively. Meanwhile, compared with MobileNetV1, MobileNetV2, ShuffleNetV1, and ShuffleNetV2 lightweight networks. The size, parameters, and FLOPs of the GMA-NET model are not only smaller, but also the model accuracy is improved by 3.86, 5.93, 5.24, and 6.1, respectively.

**TABLE 4:** Comparison of the identification results of different CNN models.

Models	Precision	Recall	F1_score	Accuracy	Size (MB)	Parameters (M)	FLOPs (M)
VGG16	85.45	85.44	85.09	84.53	1000	134	268.5
ResNet50	93.26	93.39	93.20	92.61	180	23.6	47.1
InceptionV3	93.15	93.23	93.03	92.31	167	21.8	43.6
DenseNet121	96.61	96.67	96.55	96.01	54.6	7.04	13.9
MobileNetV1	93.90	93.97	93.77	94.20	24.8	3.23	6.43
MobileNetV2	91.73	91.69	91.38	92.13	17.8	2.28	4.48
ShuffleNetV1	93.94	93.88	93.70	92.82	16.2	1.94	3.83
ShuffleNetV2	92.22	92.14	91.84	91.96	10.5	1.28	2.52
**GMA-Net**	**97.66**	**97.71**	**97.63**	**98.06**	**5.62**	**0.65**	**1.83**

In general, the GMA-Net model has a relatively small number of parameters and floating-point calculation to obtain better convergence and the highest accuracy of rubber leaf disease among the compared classical CNN model and lightweight model.

**FIGURE 6:**
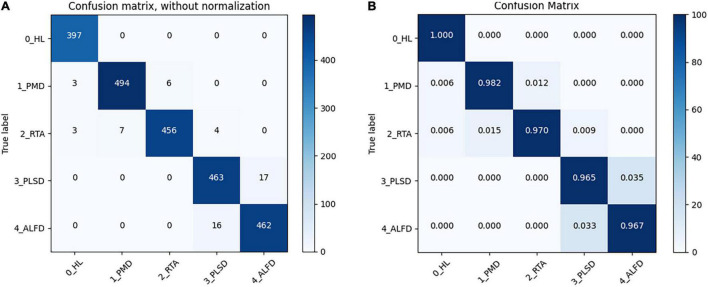
Confusion matrix of GMA-Net. **(A)** Without normalization. **(B)** Normalized (“Healthy Leaves”: 0, “Powdery Mildew Disease”: 1, “Rubber Tree Anthracnose”: 2, “Periconla Leaf Spot Disease”: 3, “Abnormal Leaf Fall Disease”: 4).

In addition, the confusion matrixes are used to summarize the performance of GMA-Net, as shown in Figure 6. The diagonals in the matrix are correctly classified, while all other entries are misclassified. It can be seen from the confusion matrix without normalization that 397 healthy leaves (0_HL), 494 Powdery Mildew Disease (1_PMD), 456 Rubber Tree Anthracnose (2_RTA), 463 Periconla Leaf Spot Disease (3_PLSD), and 462 Abnormal Leaf Fall Disease (4_ALFD) were correctly classified, and the number of misclassifications for 0_HL, 1_PMD, 2_RTA, 3_PLSD, and 4_ALFD is 0, 9, 14, 17, and 16, respectively. It can be seen from the confusion matrix that the accuracy of healthy leaves, rubber tree anthracnose, and powdery mildew disease was more than 97%, and the accuracy of periconla leaf spot disease and abnormal leaf fall disease reached 96.5 and 96.7%. Therefore, we can say that it is difficult to distinguish between periconla leaf spot disease and abnormal leaf fall disease classes.

### Ablation Experiment of Model Structure

To determine the final structure of the model, ablation experiments were carried out on the proposed model. We only retained the GMA block1 and GMA block2 mentioned in the “Network architecture” section and used them as basic models. Based on the basic model, we designed the following five combinations: GMA-Net-V1 (*N* = 1), GMA-Net-V2 (*N* = 2), GMA-Net-V3 (*N* = 3), GMA-Net-V4 (*N* = 4), and GMA-Net-V5 (*N* = 5) to test the dataset we constructed, where *N* represents the number of GMA blocks added to the basic model. The specific experimental results are shown in Table 5. In the beginning, as the number of cascaded GMAB blocks increases, the accuracy improves. For example, the recognition accuracy of GMA-Net-V1 is 97.03%. The recognition accuracy of GMA-Net V2 is 97.51%, and the GMA-Net V3 has a better effect of 98.06%, which is the highest among all comparison models. However, when the number of cascades of GMA blocks reaches 4 and 5, the accuracy of GMA-Net V4 and GMA-NET-V5 is 0.6 and 1.9% lower than that of GMA-NET-V3, and the model parameters are also improved by 0.87 and 4.31 M. The excessive number of cascaded GMA blocks may cause parameter redundancy, computational resource waste, and precision decline due to overfitting problems. If the number of cascaded GMA blocks is too small, the classification result will be unsatisfactory. In general, the appropriate number of GMAB blocks can effectively improve the accuracy of recognition but do not significantly increase the amount of computation.

**TABLE 5:** Classification results of different numbers of GMA blocks.

Models	Precision	Recall	F1_score	Accuracy	Size (MB)	Parameters (M)	FLOPs (M)
GMA-Net-V1	96.95	96.95	96.86	97.03	2.04	0.22	0.68
GMA-Net-V2	97.37	97.36	97.29	97.51	3.53	0.39	1.29
**GMA-Net-V3**	**97.66**	**97.71**	**97.63**	**98.06**	**5.62**	**0.65**	**1.83**
GMA-Net-V4	97.50	97.49	97.42	97.46	12.3	1.52	4.67
GMA-Net-V5	96.88	96.90	96.81	97.16	38.6	4.96	14.7

### Effect of Dilated Convolution and Cross-Scale Attention Feature Fusion Module

Compared with other deep learning models, this study utilizes multiple dilated convolutions with different dilation rates to extract multi-scale receptive field features and increase the model’s adaptability to the scale variability of disease spots. To verify the effect of dilated convolution on classification, all the dilated convolutions were replaced by standard convolutions, and the comparison results are shown in Table 6.

**TABLE 6:** Effect of standard and dilated convolution.

Models	Parameters (M)	F1_score					Accuracy
		0_HL	1_PMD	2_RTA	3_PLSD	4_ALFD	
Without dilated convolution	0.658	0.99	0.97	0.97	0.95	0.96	96.91
Without CAFF	0.657	0.99	0.98	0.98	0.94	0.95	96.82
Base (With dilated convolution, CAFF)	**0.658**	**0.99**	**0.98**	**0.98**	**0.96**	**0.97**	**98.06**

It can be seen that the recognition accuracy of standard convolution is 96.91%, but after replacing standard convolution with dilated convolution, the accuracy is improved from 96.91 to 98.06%, which improves the recognition accuracy of rubber leaf diseases. The reason why standard convolution shows an inferior performance is that it only samples at a fixed scale, which could not capture the scale variability of disease spots. Dilated convolution contributes to learn multi-scale useful information of disease spots and improves the recognition accuracy of the model.

In addition, we compare the classification accuracy of feature extraction with the CAFF module and without the CAFF module, respectively. It can be seen that the recognition accuracy of models without CAFF module is 96.82%, but when the CAFF module is added, the accuracy increases from 96.82 to 98.06%, which verifies the contribution of the CAFF module in classification. The CAFF module has the advantage of integrating multi-scale attention features, while reducing the influence of complex background in the image, and can provide more discriminative features.

### Visualization Results for Different Models

To better understand the learning capacity of the proposed GMA-Net model, Grad-cam (Selvaraju et al., 2016) was used to display the visualization results of different models, as shown in Table 7. The first column is disease class and the second column is the original image, followed by the visualization results of ResNet50, DenseNet121, MobileNetV2, ShuffleNetV2, and GMA-NET model in sequence. The visualization result is composed of the superposition of the rubber leaf disease image and their heatmaps. Heatmaps of ResNet50 and ShuffleNetV2 highlight the local leaf spot area, but the accuracy of heat maps was not high. Heatmaps of DenseNet121 and MobileNetV2 highlight the global leaf spot area but contain a lot of irrelevant background information. Compared with ResNet50 and DenseNet121 benchmark CNN model and MobileNetV2 and ShuffleNetV2 lightweight CNN model, the proposed GMA-NET model can accurately focus on the key areas of rubber leaf spots, with high heatmap accuracy and pays minimum attention to the irrelevant complex background, thus achieving higher disease recognition accuracy than other models.

**TABLE 7:** Visualization results of different models.

Class	Original image	ResNet50	ShuffleNetV2	DenseNet121	MobileNetV2	GMA-Net
Rubber tree anthracnose	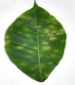	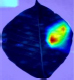	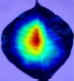	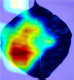	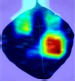	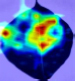
Powdery mildew disease	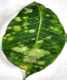	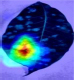	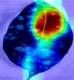	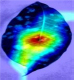	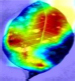	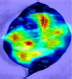
Periconla leaf spot disease	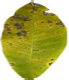	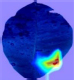	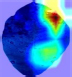	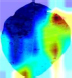	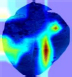	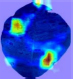
Abnormal leaf fall disease	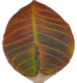	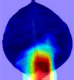	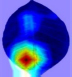	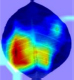	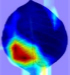	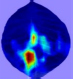

### Experiment on the Open Dataset

**FIGURE 7:**
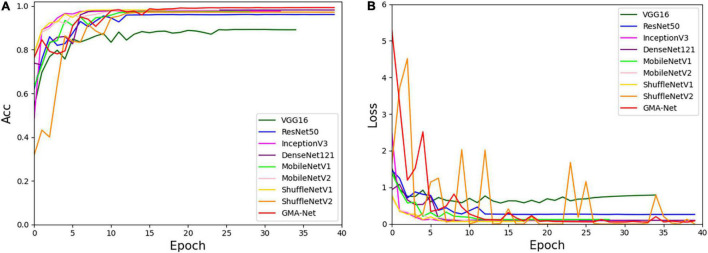
Accuracy curve and loss curve of PlantVillage validation set. **(A)** Accuracy curve. **(B)** Loss curve.

To verify the effectiveness and robustness of the proposed GMA-Net, the PlantVillage public dataset was used for verification. The PlantVillage dataset consists of 54,303 images of healthy and unhealthy leaves, divided into 38 categories by species and disease. According to the training parameters in the “Experimental configuration and hyperparameter setting” section, we divided the PlantVillage dataset into the training set, the validation set, and the test set with 32,571, 10,852, and 10,852 pictures, respectively. Then, based on the PlantVillage dataset, a comparative experiment was carried out with VGG16, ResNet50, InceptionV3, DenseNet121, MobileNetV1, MobileNetV2, ShuffleNetv1, and ShuffleNetv2. Figure 7 shows the accuracy curve and loss curve of the abovementioned eight networks and GMA-Net on the validation dataset. It can be seen from the accuracy curve that GMA-Net has the highest recognition accuracy than other models, and the loss curve shows that the loss value performed well. The test set accuracy, model size, FLOPs, parameters, top-1 accuracy, and top-5 accuracy of different models on the PlantVillage dataset are shown in Table 8.

**TABLE 8:** Results of the PlantVillage test set.

Models	Precision	Recall	F1_score	Top-1	Top-5	Parameters (M)	Size (MB)	FLOPs (M)
VGG16	86.53	85.43	89.18	89.25	98.99	134	1000	268.5
ResNet50	95.35	95.21	95.87	96.14	99.23	23.6	180	47.1
InceptionV3	96.84	96.79	97.68	97.75	99.88	21.8	167	43.6
DenseNet121	96.80	97.17	97.67	97.62	99.76	7.07	54.9	13.9
MobileNetV1	96.78	96.82	97.57	97.45	99.83	3.27	25.1	6.43
MobileNetV2	97.55	97.53	98.17	98.19	99.87	2.32	18.1	4.48
ShuffleNetV1	97.26	97.47	98.01	98.18	99.96	1.99	16.6	4.21
ShuffleNetV2	96.34	96.54	97.33	97.25	99.88	1.31	10.8	2.58
GMA-Net	**99.14**	**99.14**	**99.36**	**99.43**	**99.97**	**0.69**	**5.88**	**2.21**

Table 8 reports that the top-1 accuracy of VGG16, ResNet50, InceptionV3, DenseNet121, MobileNetV1, MobileNetV2, ShuffleNetv1, and ShuffleNetv2 is 89.25, 96.14, 97.75, 97.62, 97.45, 98.19, 98.01, and 97.25%, respectively. The top-1 accuracy rates of the GMA-Net model are 99.43%, which is the highest of all the models. In addition, the parameters, size, and FLOPs of the GMA-Net model are 0.69, 5.88, and 2.21 M, respectively, which are lower than those of other classical CNN models and lightweight models. In general, the performance of the model on the PlantVillage public dataset shows that the GMA-Net model is efficient and robust, and it is an excellent lightweight CNN network with good performance in the field of crop disease identification.

## Conclusion and Future Work

In this article, GMA-Net was proposed for rubber leaf disease image recognition. In our method, a GMDC module is responsible for multi-scale feature extraction, including small-scale fine-grained lesion features and large-scale coarse-grained lesion features. In the next phase, the CAFF module is used to fuse attention features of different scales by combining the GMDC module and the CAFF module to build the fine-grained GMA-Net model. To verify the effectiveness and robustness of the model, experiments were conducted on the constructed rubber leaf disease dataset and PlantVillage public dataset and compared with the lightweight and classical CNN models, such as ResNet50, DenseNet121, MobileNetV1, MobileNetV2, ShuffleNetV1, and ShuffleNetV2. The recognition accuracy of the model is 98.06 and 99.43%, which is the highest. In future, we collect more images of different types of rubber leaf diseases and deploy the proposed model on mobile devices.

## Data Availability Statement

The original contributions presented in the study are included in the article/Supplementary Material, further inquiries can be directed to the corresponding authors.

## Author Contributions

TZ designed and performed the experiment, selected the algorithm, analyzed the data, trained the algorithms, and wrote the manuscript. TZ, CL, BZ, and RW collected data. JW monitored the data analysis. WF and XZ conceived the study and participated in its design. All authors contributed to this article and approved the submitted version.

## Conflict of Interest

The authors declare that the research was conducted in the absence of any commercial or financial relationships that could be construed as a potential conflict of interest.

## Publisher’s Note

All claims expressed in this article are solely those of the authors and do not necessarily represent those of their affiliated organizations, or those of the publisher, the editors and the reviewers. Any product that may be evaluated in this article, or claim that may be made by its manufacturer, is not guaranteed or endorsed by the publisher.

## Supplementary Material

Supplementary Figure 1 The leaf samples were collected at the rubber tree cultivation farm in Danzhou City, Hainan Province, China. **(A)** Healthy leaves, **(B)** powdery mildew disease, **(C)** rubber tree anthracnose, **(D)** periconla leaf spot disease, and **(E)** abnormal leaf fall disease.
