# Supplementary figures and images for: Rubber Leaf Disease Recognition Based on Improved Deep Convolutional Neural Networks With a Cross-Scale Attention Mechanism
Source: Front Plant Sci. 2022 Feb 28;13:829479. doi: 10.3389/fpls.2022.829479 (PMC8918928; doi:10.3389/fpls.2022.829479)

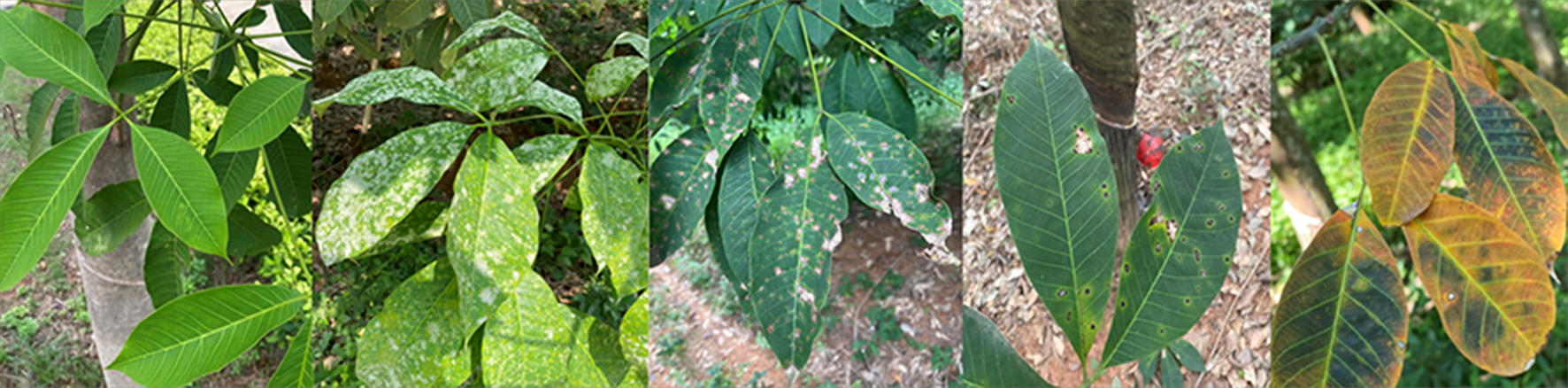

Supplement: Supplementary Figure 1 — The leaf samples were collected at the rubber tree cultivation farm in Danzhou City, Hainan Province, China. (A) Healthy leaves, (B) powdery mildew disease, (C) rubber tree anthracnose, (D) periconla leaf spot disease, and (E) abnormal leaf fall disease. [file Image_1.TIF]
